# Mini-Review on Catalytic Hydrogen Evolution from Porphyrin–Graphene Structures

**DOI:** 10.1021/acs.energyfuels.4c03322

**Published:** 2024-09-25

**Authors:** Emmanouil Nikoloudakis, Athanassios G. Coutsolelos, Emmanuel Stratakis

**Affiliations:** † Institute of Electronic Structure and Laser (IESL), Foundation for Research and Technology−Hellas (FORTH), Vassilika Vouton, 70013 Heraklion, Crete, Greece; ‡ Laboratory of Bioinorganic Chemistry, Department of Chemistry, University of Crete, Voutes Campus, 70013 Heraklion, Crete, Greece; § Qingdao Innovation and Development Center, Harbin Engineering University, Qingdao 266000 Shandong, P. R. China

## Abstract

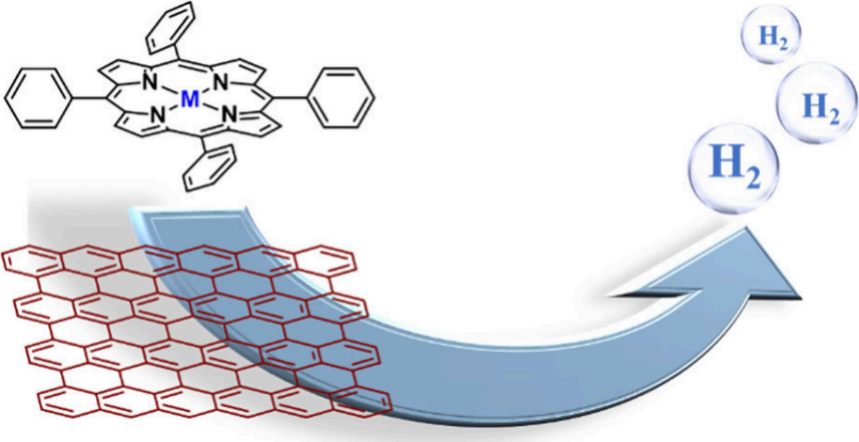

Porphyrin-based derivatives have been extensively investigated in photocatalytic, electrocatalytic, and photoelectrocatalytic H_2_ production systems as both photosensitizers and catalysts. Recently, their combination with two-dimensional materials, such as graphene oxide, reduced graphene oxide, and graphene quantum dots, has attracted significant attention for hydrogen evolution due to the advanced electronic properties, good stability, and low-cost fabrication of these materials. This mini-review summarizes the recent developments concerning the application of porphyrin–graphene ensembles in catalytic H_2_ generation. Current challenges concerning this application are discussed, and future perspectives are also proposed.

## Introduction

From the onset of the Industrial Revolution, the extraction and utilization of fossil fuels, such as coal, petroleum, and natural gas, have significantly advanced human society. Nevertheless, the escalating global energy demand has led to widespread reliance on fossil fuels, giving rise to a range of challenging environmental problems.^1^ These issues encompass global warming, acid rain, depletion of stratospheric ozone, and reduced biodiversity. Furthermore, the majority of fossil fuels fall into the category of non-renewable resources, and their reserves have undergone a substantial decline over the last decades.^2^

The escalating demand for energy along with the environmental challenges resulting from the overexploitation of fossil fuels render the imperative adoption of renewable, sustainable, and environmentally friendly energy sources. In seeking alternatives to the dependence upon fossil fuels in everyday applications, it is crucial that substitutes exhibit higher energy efficiency and, notably, lower ecological impact compared to the combustion of fossil fuels, particularly in terms of carbon emissions.^3^ Hydrogen emerges as a clean energy source with versatile applications, including automotive power, heating, fuel cells, and various others, offering a diminished environmental footprint because the only product from its combustion is molecular water.^4^ Moreover, the utilization of hydrogen as an alternative fuel represents a longstanding strategy for globally reducing carbon dioxide emissions.^5,6^

**Figure 1:**
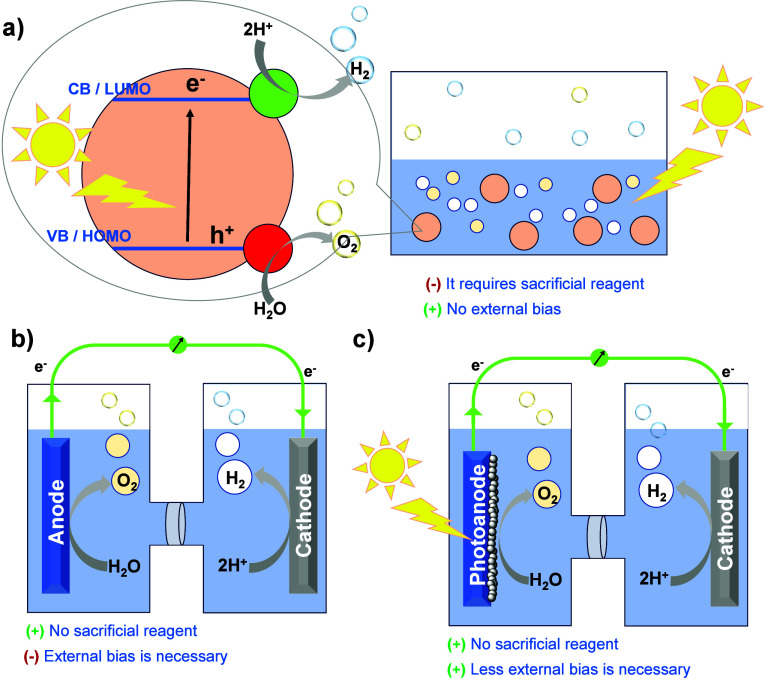
Schematic illustration of hydrogen generation from water through (a) photocatalytic, (b) electrocatalytic, or (c) photoelectrocatalytic systems.

H_2_ as an energy carrier can be produced catalytically from water through either electrocatalytic, photocatalytic, or photoelectrocatalytic schemes (Figure 1). These processes, while theoretically simple, require efficient and cost-effective catalysts to achieve practical feasibility.^7,8^ The electrocatalytic hydrogen evolution reaction (HER) comprises two distinct processes facilitating the generation of H_2_ through H^+^ reduction at catalytic sites. In both pathways, because of the applied potential, initially, H^+^ undergoes reduction to form hydride (H^–^) species at catalytic centers. Afterward, either two hydride species react, releasing molecular hydrogen, or in the second pathway, a hydride reacts with a proton–electron couple, leading to H_2_ formation.^9^

In photocatalysis, where no external potential is required, the initial step involves light absorption from the molecular or semiconductor photocatalytic component. This leads to the excitation of electrons to the lowest unoccupied molecular orbital (LUMO) or the conduction band (CB) level, respectively, and leaving holes in the highest occupied molecular orbital (HOMO)/valence band (VB) level. Efficient charge separation, redox reactions, and product desorption are crucial aspects for the HER. Optimal photocatalytic performance requires a photosensitizer with a suitable HOMO–LUMO gap/band gap for solar energy absorption, efficient charge carrier separation, and abundant active sites on the catalyst surface.^5,10^ The notable limitation of photocatalytic systems is the necessity of a sacrificial electron donor (SED) to complete the catalytic cycle. This problem can be overwhelmed by the construction of a photoelectrocatalytic device, where the necessary electrons are provided by an oxidative catalytic transformation at the photoanode, through the external circuit, to the cathode where H_2_ is generated.^11,12^ Noteworthy, this approach could be efficient in the absence of externally applied potential because the light excitation (solar energy) will be used to overcome the required energy barrier.

Nanomaterials have emerged as key elements in constructing light-energy-harvesting systems,^13,14^ offering advantages, like large surface areas and diverse morphologies. Specifically two-dimensional (2D) materials have been widely employed in green hydrogen generation systems to improve the overall efficiency and stability.^15−18^ Graphene, which stands out due to its exceptional conductivity, high surface area, optical transmittance, good stability, and low-cost fabrication, is as a versatile component for catalytic hydrogen production schemes.^6,19^ Graphene-related materials (GRMs) have already been extensively used for electrocatalytic^20−27^ and photocatalytic^28^ HER systems, displaying outstanding activity.

These materials can undergo functionalization with molecular chromophores,^29^ like porphyrins, through either covalent (i.e., amide coupling or coordination bonding) or non-covalent (i.e., π–π interactions) interactions.^30^ This strategy enhances light absorption and reduces the electron–hole recombination, improving photocatalytic performance toward H_2_ production.^10^ Additionally, it provides more active sites, i.e., metal ions with a stable coordination environment, which is beneficial for electrocatalytic H_2_ formation.^31−33^ Porphyrin molecules are particularly well-suited for this type of modification due to their easily modifiable structure, various interaction mechanisms with materials, and high molar absorption coefficient in the visible region. Finally, the porphyrin macrocycle greatly enriches the redox chemistry of the metal centers, providing improved pathways for HER catalysis.^9,34^ In electrocatalytic HER, porphyrins serve as efficient catalysts due to their ability to stabilize different oxidation states of the central metal ion, facilitating proton reduction at relatively low overpotentials.^34^ In photocatalysis, porphyrins act as light-harvesting agents, efficiently absorbing visible light and generating excited-state electrons that can drive the HER.^35^

The integration of 2D carbon materials with porphyrin-based catalysts has garnered significant attention in the field of catalytic HER. The unique electronic properties of 2D GRM offer a synergistic platform when combined with porphyrins.^36^ This synergy arises primarily from the charge transfer interactions between the delocalized π-electron systems of graphene and the π-conjugated macrocyclic structure of porphyrins.^37^ These interactions can modify the redox environment of porphyrins, thereby influencing their catalytic behavior. Understanding and controlling these charge transfer processes are crucial to optimizing the catalytic activity of these hybrid systems.

This scientific area has gained significant attention, which is illustrated by the number of existing reviews in the domain of catalytic hydrogen generation. Table 1 lists some representative reviews in the past 5 years. Most of the reviews are focused on the utilization of semiconductors, while there is no report highlighting the combination of the versatile porphyrin molecules with GRMs toward catalytic green hydrogen generation (Table 1). Therefore, the present mini-review fills this critical gap in the field by focusing on the synergistic effects between porphyrins and graphene and detailing how their combination leads to superior catalytic performance. This specific interplay and its benefits are often only briefly mentioned in other reviews that cover a wider range of materials.

**Table 1:**
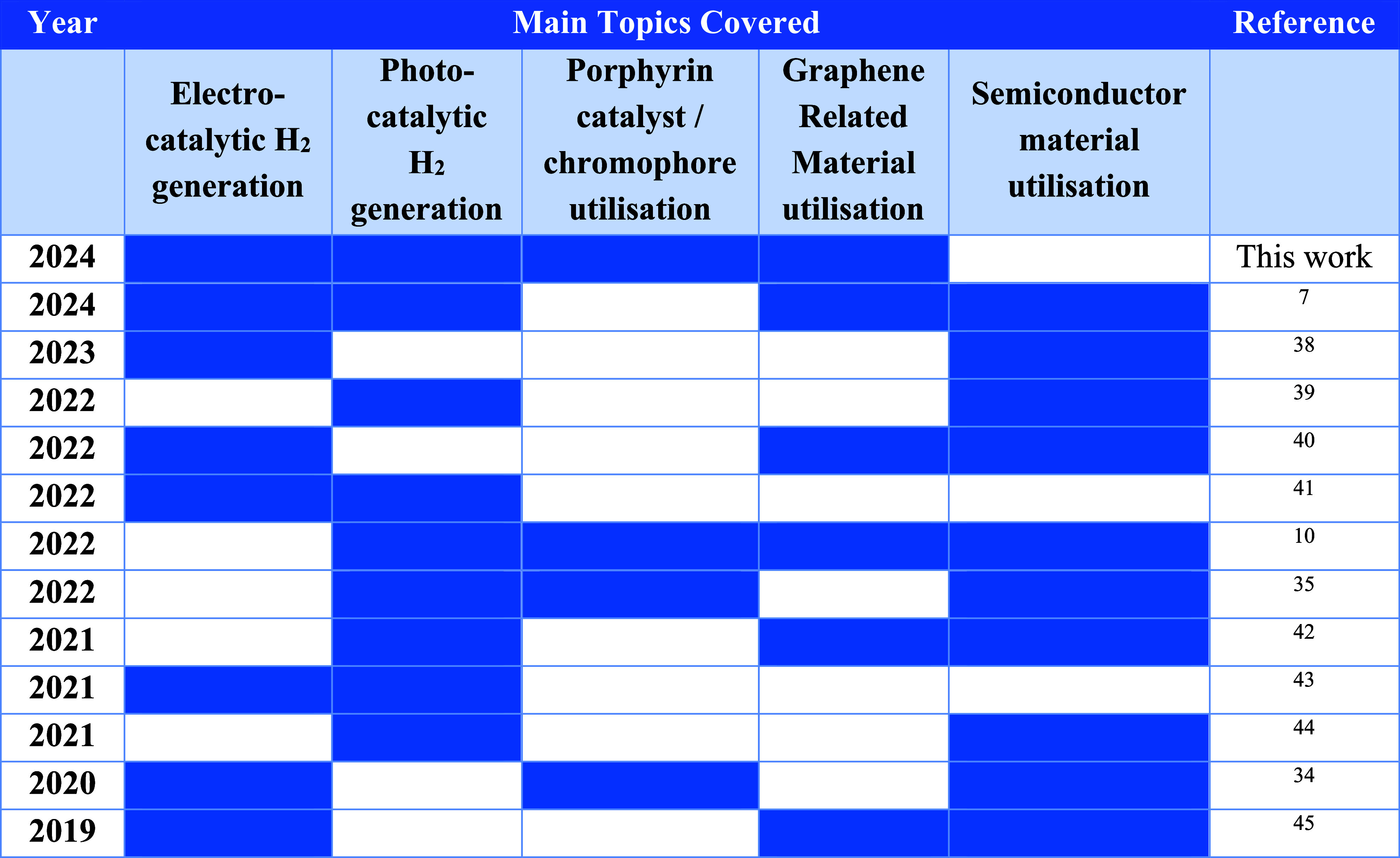
Review Studies on Electrocatalytic and Photocatalytic Hydrogen Production

Because the combination of GRM with porphyrin molecules has attracted substantial attention in sustainable energy research, this review summarizes the recent advances in electrocatalytic and photocatalytic HER from porphyrin–graphene ensembles. In the following sections, we will explore the latest advancements, the key factors that influence this catalytic transformation, the role of each catalytic component, current challenges and limitations, and promising prospects for the development of efficient HER devices. Initially, we will delve into the application of modified GRM with porphyrin molecules for electrocatalytic H_2_ evolution. Subsequently, our exploration will extend to analogous light-driven catalytic systems reported in the literature.

## Electrocatalysis

**Scheme 1:**
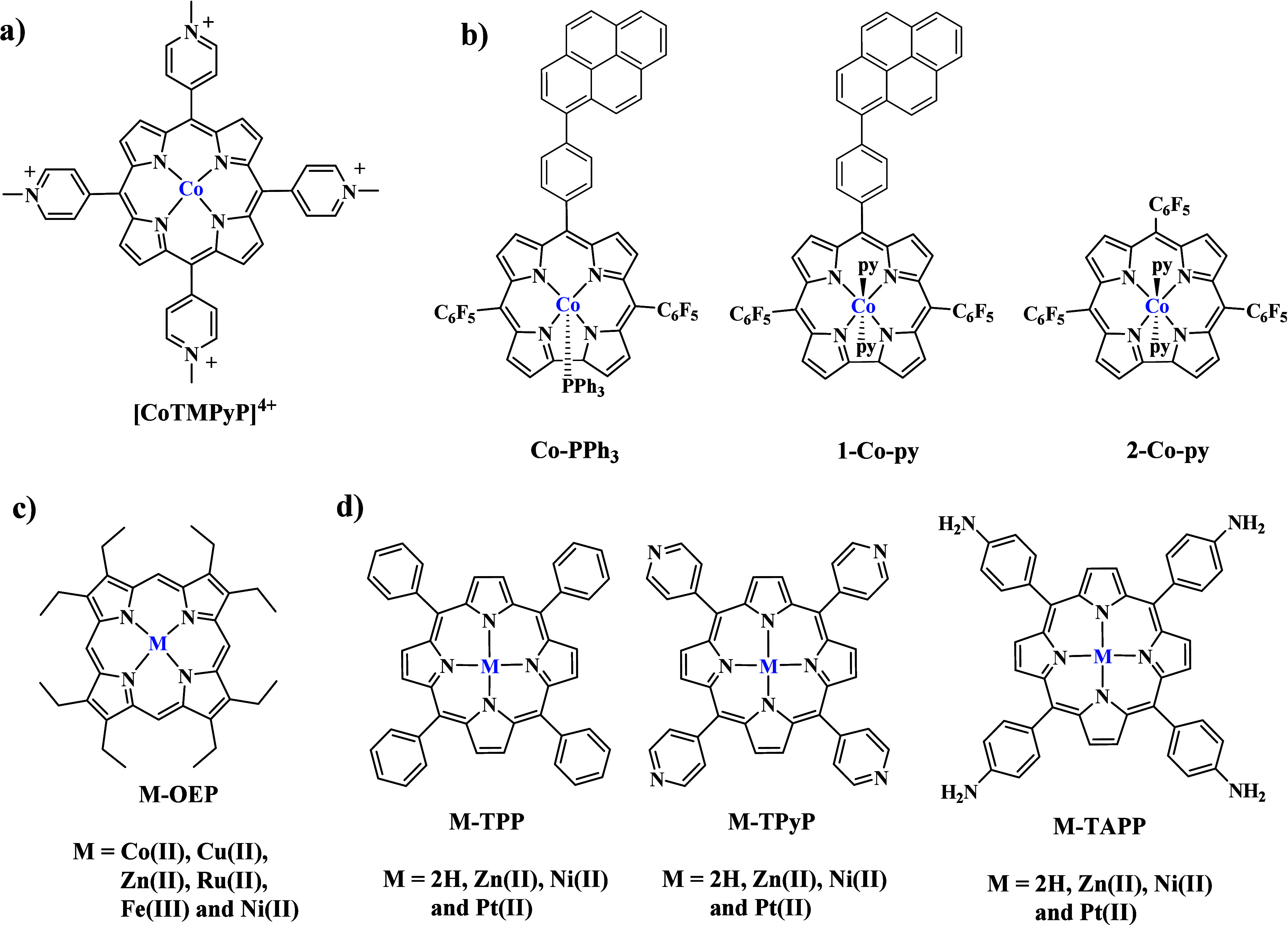
Chemical Structures of the Molecular Porphyrin Catalysts Utilized in Electrocatalytic H_2_ Evolution in Combination with GRMs

Cobalt molecular catalysts for hydrogen production have been extensively investigated during the last decades, establishing their great potential in terms of both efficiency as well as stability.^46−48^ The first system of electrocatalytic H_2_ evolution utilizing graphene oxide and a porphyrin molecular catalyst was published by Shen and co-workers and was inspired by the outstanding activity of cobalt complexes toward HER.^49^ The authors developed multilayer catalytic films composed of electrochemically reduced graphene oxide (ERGO) and positively charged cobalt porphyrin [CoTMPyP]^4+^ (Scheme 1a). The preparation of the electrodes was achieved by layer-by-layer assembly of negatively charged GO and [CoTMPyP]^4+^ via electrostatic interactions, followed by an electrochemical reduction process. Several porphyrin deposition cycles were performed before the reduction process to investigate whether this parameter affects the catalytic efficiency. The resulting multilayer electrodes were tested in electrocatalysis and presented high H_2_ production in alkaline media, while the Co–N_4_ moiety of the cobalt porphyrin proved the catalytic active site. The best performing electrode (entry 1 in Table 2) was the electrode with 7 layers of [CoTMPyP]^4+^ ([ERGO@CoTMPyP]7). Noteworthy, electrocatalytic experiments at different pH demonstrated that the pH does not influence the activity of the system because minor variations were observed from pH 0 to 13. The same cobalt porphyrin was investigated by Ma et al., who simplified the preparation protocol and further improved the electrocatalytic activity of the CoTMPyP/ERGO nanocomposite (entry 2 in Table 2).^50^

**Table 2:** Electrocatalytic Activity of Porphyrin–GRM Assemblies toward Hydrogen Evolution

entry	electrocatalyst	electrolyte	Tafel slopes (mV dec^–1^)	*E*_onset_ versus RHE (V)	current density (mA cm^–2^)	overpotential, η (mV)	FE (%)	TON/TOF	reference
1	[ERGO@CoTMPyP]7	0.1 M KOH, pH 13.0	116			474			(49)
2	CoTMPyP/ERGO	0.1 M KOH, pH 13.0	99			347			(50)
3	Co-PPh_3_/G	1.0 M KOH, pH 14.0		–0.45	166 at −0.90 V				(51)
4	1-Co-py/G	1.0 M KOH, pH 14.0		–0.45	100 at −0.90 V				(51)
5	2-Co-py/G	1.0 M KOH, pH 14.0		–0.45	73 at −0.90 V				(51)
6	GC pyridyl + Co-OEP	0.066 M phosphate buffer, pH 7.0	194				60	6400 TON	(52)
7	GC ox + Cu-OEP	0.066 M phosphate buffer, pH 7.0	103				61	3300 TON	(52)
8	GC + Cu-OEP	0.066 M phosphate buffer, pH 7.0	90				58	6500 TON	(52)
9	Fe-PMOF/PG	1.0 M KOH, pH 14.0	73.06	–0.034		154.71			(55)
10	Ni-PC[8]/ERGO	1.0 M KOH, pH 14.0		–0.020		360		27.5 mmol h^–1^ g^–1^ TOF	(56)
11	GO–PCOF	0.5 M H_2_SO_4_	241			376			(57)
12	TPP–GO–ZnPc	0.5 M H_2_SO_4_	153		10 at −0.518 V	518			(54)

Expanding the research of cobalt tetrapyrrolic catalysts on GRMs, Cao and co-workers immobilized three cobalt corrole catalysts on graphene and applied the resulting materials in electrocatalytic hydrogen evolution in aqueous media.^51^ In detail, they synthesized corrole molecules Co-PPh_3_, 1-Co-py, and 2-Co-py (Scheme 1b) and supported them on graphene to obtain catalytically active materials toward H_2_ production, operating in different pH values (pH 0–14). Electrocatalysis results revealed the following performance trend: Co-PPh_3_/G > 1-Co-py/G > 2-Co-py/G, demonstrating the significant role of the axial coordinating ligand as well as the pyrenyl peripheral substitution (entries 3–5 in Table 2).

The role of the metal ion inside the porphyrin core toward electrocatalytic H_2_ evolution was investigated by Galo and co-workers.^52^ The researchers prepared glassy carbon (GC) electrodes, introduced oxo and pyridyl groups on their surfaces, and subsequently deposited octaethyl-metalated porphyrins (M-OEP; Scheme 1c). The electrodes were able to produce H_2_ electrocatalytically at pH 7, while Co-OEP and Cu-OEP presented the highest activity (entries 6–8 in Table 2). Atomic force microscopy (AFM) and scanning electron microscopy (SEM) studies showed another significant parameter that influences the catalysis, namely, the formation of appropriate supramolecular architectures on the surface of the electrode, which enables proton diffusion electron transfer processes.

Lee and co-workers developed 2D assemblies of various metalated porphyrins on graphene to shed light into the molecular aspects that govern the mechanism of electrocatalytic H_2_ production.^53^ More specifically, they investigated the tetraphenyl, tetrapyridyl, and tetraaminophenyl porphyrin molecules illustrated in Scheme 1d toward HER after their immobilization onto one layer of graphene. The authors point out the significance of the intermolecular H bonding between porphyrin molecules, the π-electron delocalization on the porphyrin macrocycle, and the electronegativity of the central N4 atoms, which assist the charge transfer to graphene. The Pt-metalated derivative presented the highest activity for H_2_ evolution in all cases, while the peripheral substitution followed the trend: phenyl < pyridyl < aminophenyl. Between the M–porphyrin/graphene assemblies, the HER performance followed the order Zn < Ni < Pt.

In another approach, Wang et al. synthesized a hybrid material consisting of TAPP porphyrin units and zinc phthalocyanine units covalently linked to GO and applied this material in electrocatalytic H_2_ generation. The three-component system was proven more efficient compared to the two-component hybrids (TPP–GO and ZnPc–GO), and this result was attributed to the strong electronic communication and charge transfer phenomena within the TPP–GO–ZnPc derivative. The authors point out the importance of simultaneous grafting of porphyrins and phthalocyanines onto GO as a promising approach for efficient electrocatalytic HER systems.^54^

**Figure 2:**
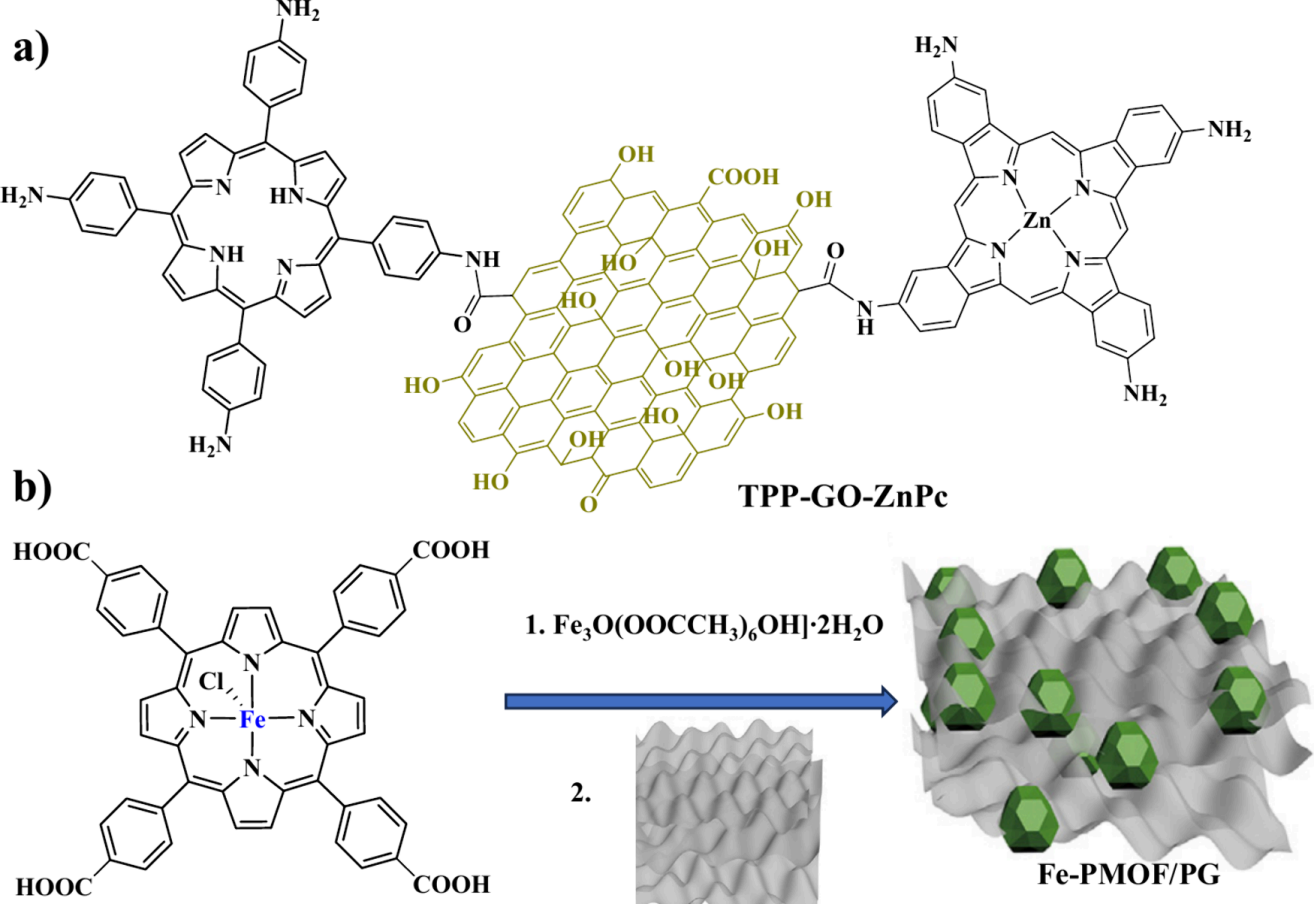
Schematic illustration for the preparation of (a) TPP–GO–ZnPc^54^ and (b) Fe-PMOF/PG. This figure was reproduced with permission from ref (55). Copyright 2018 Elsevier.

The research works described above deal with molecular HER catalytic moieties based on porphyrin derivatives, which were used in combination with 2D GRMs. There are few publications were 2D or three-dimensional (3D) porphyrin materials^58^ were utilized instead. Guo and co-workers prepared a 3D porphyrin–metal–organic framework (PMOF) based on iron tetra-carboxyphenyl porphyrin (Fe-PMOF) and, subsequently, introduced porous graphene via hydrothermal procedure, obtaining Fe-PMOF/PG (Figure 2).^55^ The researchers demonstrated that the utilization of the graphene substrate was beneficial to achieve a more stable, more conductive, and more electrocatalytically efficient material (entry 9 in Table 2).

**Scheme 2:**
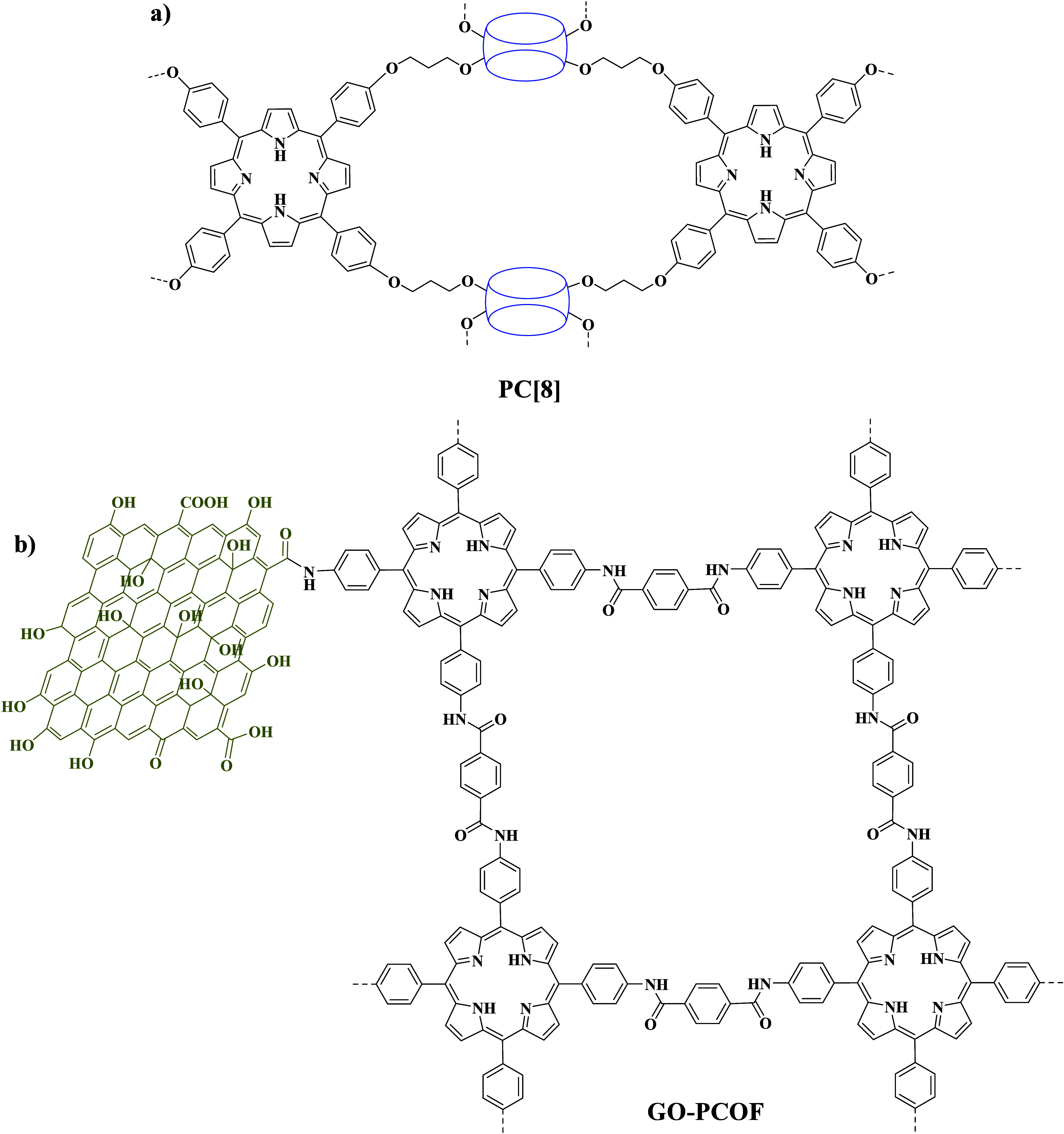
Chemical Structures of the Porphyrin Polymer Networks PC[8]^56^ and GO–PCOF,^57^ Utilized as Catalysts in Electrocatalytic H_2_ Evolution

Another type of 2D porphyrin material utilized toward HER application was porphyrin polymers. Tuncel and co-workers constructed a polymer network (PC[8]; Scheme 2a) via cross-linking tetra-aryl-substituted porphyrins and hydroxy-substituted cucurbit[8].^56^ Subsequently, the researchers loaded nickel atoms on this network, coated GO, which was deposited on FTO, with Ni-PC[8], and reduced GO afterward electrochemically. The prepared electrodes varied in terms of Ni and reduced GO mass ratio versus the PC[8] network and were proven efficient electrocatalysts for hydrogen evolution (entry 10 in Table 2). In another report, covalent coupling of the 2D porphyrin polymer with GO enhanced the HER activity. Wang et al. synthesized the porphyrin covalent organic framework (PCOF; Scheme 2b) and covalently connected graphene oxide via amide bond formation utilizing the DCC coupling reagent.^57^ The prepared GO–PCOF conjugate presented superior electrocatalytic HER performance (entry 11 in Table 2) compared to GO, PCOF, or molecular Η_2_-TPP, demonstrating the influence of the covalent attachment to the overall catalytic activity.

The recent publications reviewed above dealing with electrocatalytic H_2_ production from porphyrin–graphene ensembles have pointed out the significant factors that influence this catalytic transformation. These factors include

- the metal ion inside the porphyrin macrocycle, which in most cases is the catalytic center
- the coordination sphere of the metal porphyrin center
- the peripheral substitution of the molecular porphyrin catalyst
- the electronic communication between the π systems of GRM and the porphyrin macrocycles
- the formation of 2D or 3D porphyrin materials and the subsequent incorporation of GRMs (i.e., PMOF and PCOF)
- the different oxidation states of GRMs, namely, GO, ERGO, etc.
- the electrolyte and pH of the reaction
- the surface supramolecular architectures of the electrode

## Photocatalysis

The catalytic transformation of protons to molecular H_2_ can be boosted via either the assistance of an applied overpotential or the utilization of solar energy. This paragraph reviews the publications targeting photocatalytic H_2_ evolution from porphyrin photosensitizers/catalysts and GRMs.

**Scheme 3:**
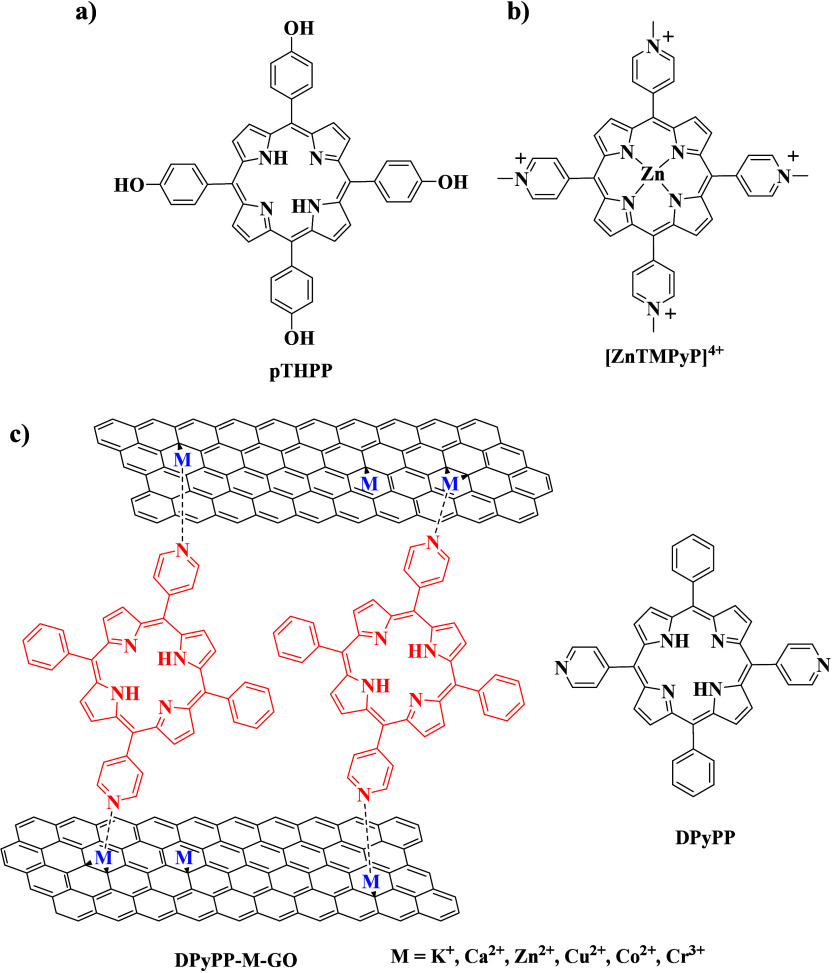
Chemical Structures of (a) pTHPP,^59^ (b) [ZnTMPyP]^4+^,^60^ and (c) DPyPP^61^ Porphyrin Derivatives Utilized as Photosensitizers for the Photocatalytic Transformation of Protons to Molecular Hydrogen DPyPP–M–GO is a schematic illustration of graphene oxide implanted with metal ions and linked through the DPyPP chromophore molecules.

The first report on this field was published by Yang and co-workers in 2013, who immobilized tetra-hydroxy porphyrin pTHPP (Scheme 3a) on rGO and investigated the morphological and electronic properties of the resulting hybrid material.^59^ Subsequently, the researchers deposited Pt NPs on the pTHPP/rGO hybrid and applied it in light-driven H_2_ evolution using triethylamine as the SED. The important role of reduced graphene oxide as an electron mediator, preventing photoinduced electron–hole recombination, was established. In an effort to increase both the stability and the efficiency of this system, a surfactant molecule (CTAB) was introduced, and indeed, it was proven beneficial toward H_2_ production (entry 1 in Table 3).

**Table 3:** Photocatalytic Activity of Porphyrin–GRM Assemblies toward Hydrogen Production

entry	PS/photocatalyst	catalyst	SED	solvent	light source	irradiation time (h)	TON/TOF	reference
1	pTHPP/rGO	Pt NPs	TEA (10 vol %)	H_2_O (CTAB)	150 W Xe lamp	5	11.2 mmol g^–1^ TOF	(59)
2	[ZnTMPyP]^4+^/MoS_2_/RGO		TEOA	H_2_O	300 W Xe lamp		2.56 mmol g^–1^ h^–1^ TOF	(60)
3	GO		TEOA (10 vol %)	H_2_O	300 W Xe lamp	8	137 μmol g^–1^ TOF	(61)
4	TPPy–GO		TEOA (10 vol %)	H_2_O	300 W Xe lamp	8	686 μmol g^–1^ TOF	(61)
5	DPyP–-Cr^(III)^–GO		TEOA (10 vol %)	H_2_O	300 W Xe lamp	8	928 μmol g^–1^ TOF	(61)
6	DPyP–Co^(II)^–GO		TEOA (10 vol %)	H_2_O	300 W Xe lamp	6	1677 μmol g^–1^ TOF	(62)
7	DPyP–Co–GO		TEOA (10 vol %)	H_2_O	300 W Xe lamp	2	1093 μmol g^–1^ TOF	(63)
8	GO–Sm^(III)^–DPyP		TEOA (16.7 vol %)	H_2_O	300 W Xe lamp	8	4.39 mmol g^–1^ TOF	(64)
9	PCOF-1	Pt/rGO	EDTA (0.5 M)	H_2_O	320 W SolarSim, λ > 420 nm		0.75 μmol h^–1^ TOF	(65)
10	exfoliated PCOF-1	Pt/rGO	EDTA (0.5 M)	H_2_O	320 W SolarSim, λ > 420 nm		1.25 μmol h^–1^ TOF	(65)
11	TPP–GO	Fe-Cat1	cystine (1.8 mM)	EtOH/water (1:24), pH 1.5	Hg lamp (450 W) (λ > 380 nm)	5	1.61 TON	(66)
12	TPP–GO	Fe-Cat2	cystine (1.8 mM)	EtOH/water (1:24), pH 1.5	Hg lamp (450 W) (λ > 380 nm)	5	1.80 TON	(66)
13	TPP–GO	Fe-Cat3	cystine (1.8 mM)	EtOH/water (1:24), pH 1.5	Hg lamp (450 W) (λ > 380 nm)	5	2.82 TON	(66)
14	TPP–GO	Ni-Cat1	AA (0.1 M)	EtOH/water (1:24), pH 4.0	Hg lamp (450 W) (λ > 380 nm)	5	1.2 μL of H_2_	(67)
15	TPP–GO		TEA (10 vol %)	H_2_O	UV–vis	6	3.8 μmol mg^–1^ TOF	(68)
16	TPP–GO	Pt	TEA (10 vol %)	H_2_O	UV–vis	6	4.6 μmol mg^–1^ TOF	(68)
17	TPP–GO/PVP	Pt	TEA (10 vol %)	H_2_O	UV–vis	6	5.2 μmol mg^–1^ TOF	(68)
18	GO–pTHPP–PSA		TEA	H_2_O	λ = 450 nm	8	44.3 μmol g^–1^ h^–1^ TOF	(69)
19	GQ-dots/Pt–pTHPD		AA (0.1 M)	H_2_O	λ > 420 nm		57.19 mmol g^–1^ h^–1^ TOF	(71)
20	rGO/g-C_3_N_4_/Cu/TCPP nanorods		TEOA (20 vol %)	H_2_O	300 W Xe lamp	5	4 mmol g^–1^ TOF	(73)

In another report, Yuan et al. used water-soluble porphyrin [ZnTMPyP]^4+^ (Scheme 3b) as a light-absorbing moiety together with 2D material MoS_2_/rGO as the catalytic moiety and developed a heterogeneous system for efficient H_2_ production.^60^ Noteworthy, this is a rare photocatalytic example where the photosensitizer and catalyst are in different states (solution versus dispersion). Upon visible light irradiation and in the presence of TEOA as the SED, the system reached 2560 μmol g^–1^ h^–1^ of H_2_ production (entry 2 in Table 3). Mechanistical investigation demonstrated that, after light excitation, the porphyrin–chromophore is quenched by MoS_2_/rGO following an oxidative quenching.

An alternative approach for the introduction of porphyrins on the surface of graphene oxide involves attaching metal ions to GO, which can serve as interfacial linkers. The attachment of porphyrin molecules can be achieved via either electrostatic interactions or coordination bonding. For this purpose, several metal cations (K^(I)^, Ca^(II)^, Zn^(II)^, Cu^(II)^, Co^(II)^, and Cr^(III)^) were implanted onto graphene oxide forming the M–GO material.^61^ The subsequent introduction of DPyPP porphyrin (Scheme 3c) was accomplished by simply mixing modified graphene oxide with the DPyPP chromophore. The researchers continued with the photocatalytic investigation toward H_2_ production using triethylamine as the SED and showed that the catalytic activity follows the order GO < DPyPP-GO < DPyPP–Cr^(III)^–GO (entries 3–5 in Table 3). The high performance of the DPyPP–Cr^(III)^–GO photocatalyst was attributed to the strong coordination interaction between metal ions and porphyrin, while the role of the porphyrin molecule was to absorb the visible light, and the graphene oxide served as the electron mediator. The metal ions were proven beneficial for the promotion of the photogenerated electrons, thus improving the overall performance. The same group further explored this field with the utilization of another metal ion, namely, Co^(II)^, and prepared the DPyPP–Co^(II)^–GO photocatalytic material (Scheme 3c).^62^ The photocatalyst was able to produce H_2_ without the need of other co-catalysts, outperforming the previous systems (entry 6 in Table 3). The researchers verified that Co^(II)^ ions promote electron propagation between the porphyrin moiety and graphene oxide, improving the overall activity of the system. With the above results in hand, the same group prepared DPyPP–Co–GO, which, instead of cobalt ions, contained metallic Co nanoparticles, with the aim to achieve further improved catalytic performance because of the co-catalytic function of the NPs.^63^ Indeed, the observed visible-light-driven H_2_ evolution of DPyPP–Co–GO was doubled in comparison to DPyPP–Co^(II)^–GO after 2 h of irradiation (entry 7 in Table 3). The exploration of rare earth metal ions intrigued the researchers to implant Sm^(III)^ on the surface of graphene oxide, and therefore, they developed and studied the DPyPP–Sm^(III)^–GO photocatalytic material.^64^ The interaction of Sm^(III)^ with the oxygen species of graphene oxide and its coordination with the pyridyl peripheral substitution of DPyPP porphyrin were demonstrated. Photocatalytic investigation showed that the DPyPP–Sm^(III)^–GO hybrid reached a H_2_ production activity of 4.39 mmol/g after 8 h of visible light irradiation (entry 8 in Table 3), pointing out the advantageous role of Sm^(III)^ to the stability and efficiency of the catalytic system.

**Scheme 4:**
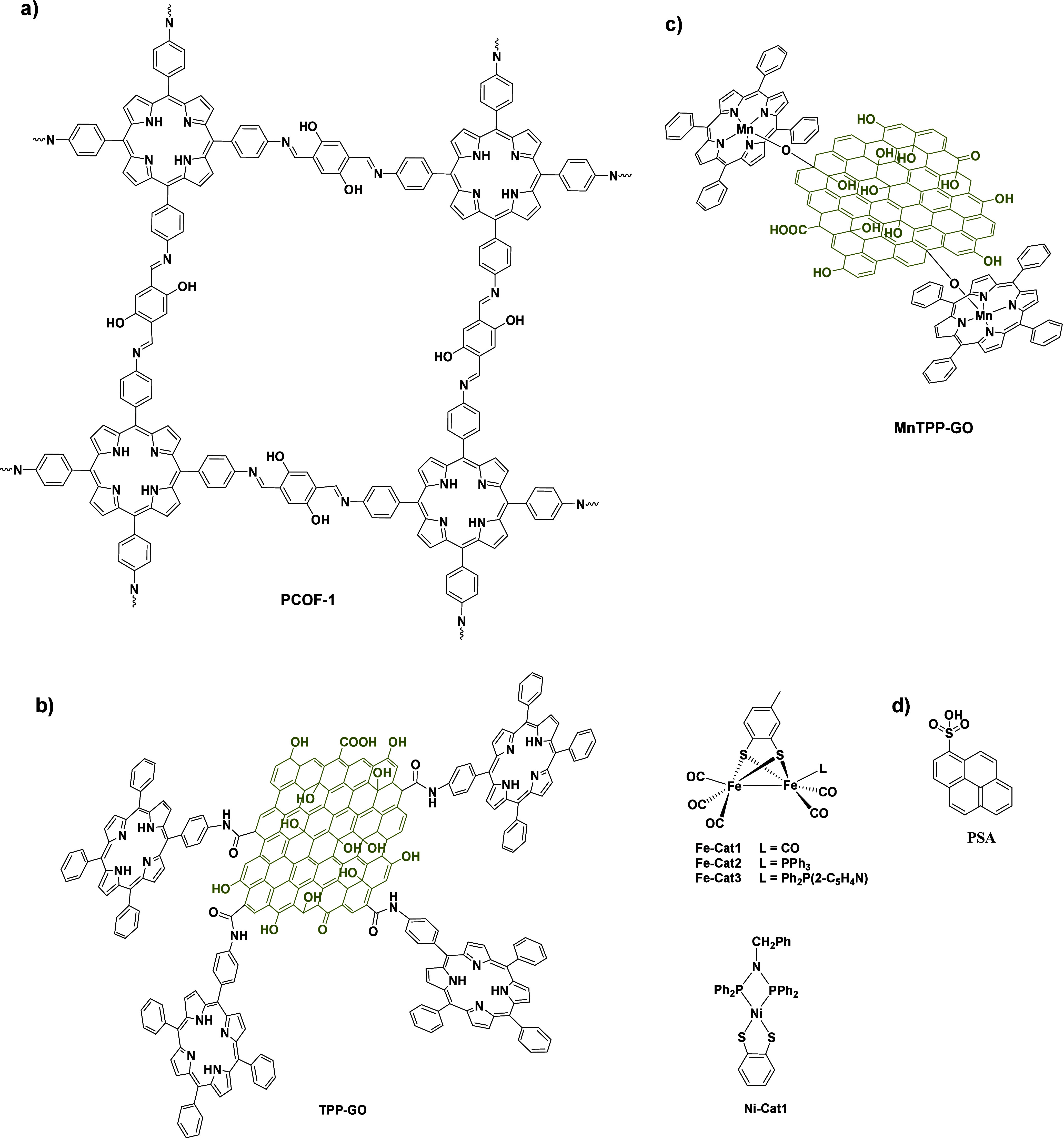
Chemical Structures of (a) Porphyrin Organic Framework PCOF Used by Fan et al.,^65^ (b) TPP–GO Used as a Photosensiter in Combination with a [Fe–Fe]-Hydrogenase Model or Ni–Cat1 Complex as Catalysts,^66,67^ and (c) Mn–TPP–GO Photocatalyst^68^

Fan et al. developed 2D nanodisks comprising exfoliated PCOF-1 (Scheme 4a) through the incorporation of Mg^2+^ and Cu^2+^ ions as well as appropriate axial ligands.^65^ The researchers tested the starting PCOF material and the exfoliated nanodisks in visible-light-driven H_2_ evolution, with the latter demonstrating higher activity. The introduction of a co-catalyst material, namely, Pt/rGO, further improved the catalytic efficiency probably due to high surface interactions between the 2D PCOF nanodisks and the 2D Pt/rGO co-catalyst (entries 9 and 10 in Table 3).

In another report, Li et al. covalently connected GO with tetraphenyl porphyrin via amide coupling to obtain the photosensitizing entity TPP–GO (Scheme 4b), which was used in combination with [Fe–Fe]-hydrogenase models (Scheme 4b) as catalytic entities.^66^ The researchers showed the non-covalent interactions between the catalyst and 2D TPP–GO in aqueous ethanol solution and continued with establishing the catalytic activity of the Fe complexes electrocatalytically. They investigated the photocatalytic activity of the system toward H_2_ production utilizing cysteine as the SED and revealed the role of the coordinating ligands on the Fe–Fe catalyst (entries 11–13 in Table 3) as well as the importance of the porphyrin moiety for the absorption in the visible region. Covalent linkage between GO and porphyrin was proven beneficial for the catalytic performance, while GO was also proven essential in enhanced H_2_ production, serving as both an electron mediator and a scaffold. This system was further improved by the same research group, who applied TPP–GO as a light-absorbing component and Ni complex (Scheme 4b) as a catalyst in EtOH/H_2_O solution using ascorbic acid as the SED (entry 14 in Table 3).^67^ The researchers verified again the importance of all components toward H_2_ generation and demonstrated the enhancement of the catalytic activity due to the presence of GO. Besides amide coupling, which was explored in the previous example, GO can be linked with metalated porphyrins through the axial coordination with the metal center. In this concept, Yang and co-workers reported the connection of Mn–TPP porphyrin with GO (Scheme 4c) and used the resulting material in light-driven H_2_ production with triethylamine serving as the SED and Pt NPs as catalytic moieties (entries 15–17 in Table 3).^68^ Aggregation was proven a limiting factor in the overall performance of the system and was overcome with the addition of surfactants (PVP, CTAB, and SDS), with non-ionic PVP showing the best results.

Another approach was presented by Li and co-workers, who employed two chromophores to achieve broader photosensitizing ability.^69^ More specifically, the authors grafted pTHPP (Scheme 3a) and pyrene sulfonic acid (PSA; Scheme 4d) onto the surface of graphene oxide utilizing π–π stacking and hydrogen-bonding interactions. The synthesized three-component 2D material, GO–pTHPP–PSA, displayed enhanced photocatalytic H_2_ evolution in comparison to the catalytic systems with one or more components absent (entry 18 in Table 3).

**Scheme 5:**
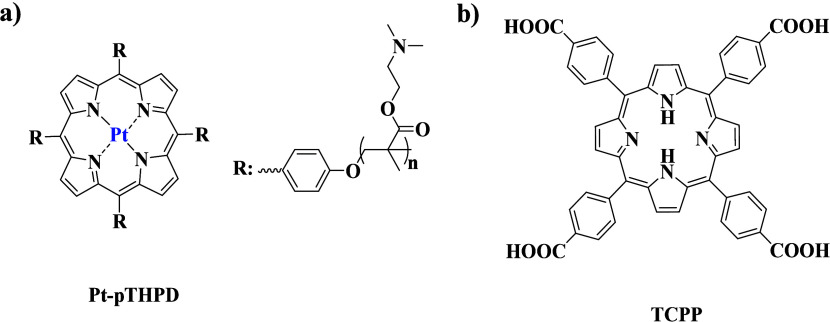
Chemical Structures of (a) Pt–pTHPD Porphyrin Star Polymer^71^ and (b) TCPP Used by Li et al.^73^

Another interesting type of GRM is graphene quantum dots (GQ-dots),^70^ and they were applied in photocatalytic H_2_ production in combination with a porphyrin star polymer by Ji et al.^71^ More specifically, the authors synthesized polymeric Pt–pTHPD (Scheme 5a) as a catalytic moiety, known for its ability to form micelles^72^ as well as GQ-dots antenna light-harvesting-materials according to a solvothermal procedure. Subsequently, the positively charged Pt–pTHPD micelles self-assembled via electrostatic interactions with the negatively charged GQ-dots and formed a supramolecular photocatalyst GQ-dots/Pt–pTHPD, which reached a very high H_2_ production rate under visible light irradiation and displayed enhanced stability (entry 19 in Table 3). One of the latest examples of GRMs and porphyrins applied in the catalytic transformation of protons to molecular hydrogen was reported by Li and co-workers.^73^ The researchers developed a 2D material consisting of a reduced graphene oxide (rGO)–graphitic carbon nitride (g-C_3_N_4_) nanocomposite implanted with Cu nanoparticles and self-assembled TCPP (Scheme 5b) nanorods. The cooperative effect of rGO and Cu nanoparticles was proven to be the main reason for the enhanced H_2_ evolution that was achieved (entry 20 in Table 3).

The reports reviewed above dealing with photocatalytic H_2_ production from porphyrin–graphene ensembles have pointed out the important factors that govern this catalysis, additional to the factors already discussed in the electrocatalytic section. These factors include

- the nature of the porphyrin (i.e., molecular chromophore, polymer, and PCOF)
- the peripheral substitution and metal coordination of the porphyrin, which in most reports is the photosensitizing moiety
- the linkage and interactions (electrostatic and π–π stacking) between the porphyrin molecules and the GRM
- the utilization of appropriate molecules of nanoparticle catalysts and co-catalyst
- the photocatalytic reaction conditions (solvent, pH, and light source)

## Challenges and Future Perspectives

Porphyrins have already been extensively investigated in photo-, electro-, and photoelectrocatalytic H_2_ production schemes, while recently, their combination with 2D graphene-based materials has attracted extended scientific attention. The two fundamental remaining challenges include the need for further increased performance and further improved stability to beat commercial processes, like steam methane reforming and electrolysis. GO, rGO, and GQ-dots offer their advanced electronic properties, good stability, and low-cost fabrication, while porphyrins retain their inherent photophysical and electrochemical characteristics; therefore, the resulting hybrid materials have enhanced activity and prolonged lifespan. This strategy was proven very efficient; thus, other chromophores with alike photophysical features, such as corroles, phthalocyanines, and BODIPY dyes, could also be used as H_2_ production catalysts. While porphyrinoids offer significant advantages toward HER, they also have certain limitations, including their susceptibility to degradation under prolonged catalytic conditions due to metal leaching or macrocycle photodegradation. To address the aspect of catalytic stability in the practical application of chromophore–GRM assemblies toward HER, there are strategies such as the incorporation of stabilizing ligands and enhanced binding groups between porphyrinoid and the GRM, which can significantly improve the extended activity.

Moving one step forward, we suggest as future directions the development of dye-sensitized photoelectrochemical cells (DSPECs) utilizing porphyrin–graphene hybrid materials. This approach could advance the overall performance toward H_2_ evolution, and it is one step closer to a real-world application because it requires no sacrificial reagents and no externally applied potential. To the best of our knowledge, there is still no report following this strategy for hydrogen generation, while publications concerning other reductive catalytic transformations (i.e., CO_2_ reduction) have already been reported.^74^ Noteworthy, other types of 2D materials (i.e., g-C_3_N_4_) are commonly studied in both photocatalysis and photocurrent response, demonstrating the potential for large-scale implementation.^75^ The combination of traditional steam methane reforming and light-driven hydrogen production is also a promising avenue to follow utilizing porphyrin–graphene hybrid materials.^76^

Another interesting path to investigate would be the utilization of porphene,^77,78^ the porphyrin analogue of graphene, which has only recently been synthesized, exhibits interesting properties, and is still unexplored in hydrogen generation catalysis. Noteworthy, similar 2D materials have been proposed via computational studies as promising single-atom electrocatalysts.^79^ The synthesis of 2D porphene and first raw metalated porphene is expected to open new directions in this field due to the robustness, visible light absorption ability, and catalytic productivity of porphyrin units. One critical area for investigation involves the scalability of such materials. Current methods have been proven efficient at the laboratory scale, but the development of cost-effective, large-scale synthetic routes is crucial for the commercial exploitation of these materials.

## Conclusion

In the past decade, significant advances have been made in catalytic H_2_ production, with the aim of shifting from a fossil-fuel-based economy to a hydrogen economy. This review summarized the recent advances in electrocatalytic and photocatalytic H_2_ generation from porphyrin–graphene ensembles. After reviewing all recent publications dealing with electrocatalytic and photocatalytic H_2_ production from porphyrin–graphene nanomaterials, we pointed out the important factors that influence this catalytic transformation as well as the role of each catalytic component. The synergy between porphyrin and graphene components resulted in enhanced HER catalytic activity, stability, and efficiency, making them promising candidates for sustainable hydrogen generation. While challenges remain, particularly in scaling up production and ensuring long-term stability, the future prospects of these materials are bright.

## References

[ref1] Dalle K. E.; Warnan J.; Leung J. J.; Reuillard B.; Karmel I. S.; Reisner E. Electro- and Solar-Driven Fuel Synthesis with First Row Transition Metal Complexes. Chem. Rev. 2019, 119 (4), 2752–2875. 10.1021/acs.chemrev.8b00392. 30767519 PMC6396143

[ref2] Huang H.; Yan M.; Yang C.; He H.; Jiang Q.; Yang L.; Lu Z.; Sun Z.; Xu X.; Bando Y.; Yamauchi Y. Graphene Nanoarchitectonics: Recent Advances in Graphene-Based Electrocatalysts for Hydrogen Evolution Reaction. Adv. Mater. 2019, 31 (48), e1903415 10.1002/adma.201903415. 31496036

[ref3] Reza M. S.; Ahmad N. B. H.; Afroze S.; Taweekun J.; Sharifpur M.; Azad A. K. Hydrogen Production from Water Splitting through Photocatalytic Activity of Carbon-Based Materials. Chem. Eng. Technol. 2023, 46 (3), 420–434. 10.1002/ceat.202100513.

[ref4] Le Goff A.; Artero V.; Jousselme B.; Tran P. D.; Guillet N.; Métayé R.; Fihri A.; Palacin S.; Fontecave M. From Hydrogenases to Noble Metal-Free Catalytic Nanomaterials for H_2_ Production and Uptake. Science 2009, 326 (5958), 1384–1387. 10.1126/science.1179773. 19965754

[ref5] Cao S.; Yu J. Carbon-based H_2_-production photocatalytic materials. J. Photochem. Photobiol., C 2016, 27, 72–99. 10.1016/j.jphotochemrev.2016.04.002.

[ref6] Xie G.; Zhang K.; Guo B.; Liu Q.; Fang L.; Gong J. R. Graphene-based materials for hydrogen generation from light-driven water splitting. Adv. Mater. 2013, 25 (28), 3820–39. 10.1002/adma.201301207. 23813606

[ref7] Kumar R.; Singh R.; Dutta S. Review and Outlook of Hydrogen Production through Catalytic Processes. Energy Fuels 2024, 38 (4), 2601–2629. 10.1021/acs.energyfuels.3c04026.

[ref8] Song H.; Luo S.; Huang H.; Deng B.; Ye J. Solar-Driven Hydrogen Production: Recent Advances, Challenges, and Future Perspectives. ACS Energy Lett. 2022, 7 (3), 1043–1065. 10.1021/acsenergylett.1c02591.

[ref9] Zhang W.; Lai W.; Cao R. Energy-Related Small Molecule Activation Reactions: Oxygen Reduction and Hydrogen and Oxygen Evolution Reactions Catalyzed by Porphyrin- and Corrole-Based Systems. Chem. Rev. 2017, 117 (4), 3717–3797. 10.1021/acs.chemrev.6b00299. 28222601

[ref10] Nikoloudakis E.; López-Duarte I.; Charalambidis G.; Ladomenou K.; Ince M.; Coutsolelos A. G. Porphyrins and phthalocyanines as biomimetic tools for photocatalytic H_2_ production and CO_2_ reduction. Chem. Soc. Rev. 2022, 51 (16), 6965–7045. 10.1039/D2CS00183G. 35686606

[ref11] Idriss H. Hydrogen production from water: Past and present. Curr. Opin. Chem. Eng. 2020, 29, 74–82. 10.1016/j.coche.2020.05.009.

[ref12] Charisiadis A.; Glymenaki E.; Planchat A.; Margiola S.; Lavergne-Bril A.-C.; Nikoloudakis E.; Nikolaou V.; Charalambidis G.; Coutsolelos A. G.; Odobel F. Photoelectrochemical properties of dyads composed of porphyrin/ruthenium catalyst grafted on metal oxide semiconductors. Dyes Pigm. 2021, 185, 108908 10.1016/j.dyepig.2020.108908.

[ref13] Soultati A.; Tountas M.; Fakharuddin A.; Skoulikidou M.-C.; Verykios A.; Armadorou K.-K.; Tzoganakis N.; Vidali V. P.; Sakellis I.; Koralli P.; Chochos C. L.; Petsalakis I.; Nikoloudakis E.; Palilis L. C.; Filippatos P.-P.; Argitis P.; Davazoglou D.; Mohd Yusoff A. R. b.; Kymakis E.; Coutsolelos A. G.; Vasilopoulou M. Defect passivation in perovskite solar cells using an amino-functionalized BODIPY fluorophore. Sustainable Energy Fuels 2022, 6 (10), 2570–2580. 10.1039/D2SE00384H.

[ref14] Kundu S.; Patra A. Nanoscale Strategies for Light Harvesting. Chem. Rev. 2017, 117 (2), 712–757. 10.1021/acs.chemrev.6b00036. 27494796

[ref15] Sarkar A. S.; Stratakis E. Recent Advances in 2D Metal Monochalcogenides. Adv. Sci. 2020, 7 (21), 2001655 10.1002/advs.202001655. PMC7610304 33173730

[ref16] Chandrappa S.; Murthy D. H. K.; Reddy N. L.; Babu S. J.; Rangappa D.; Bhargav U.; Preethi V.; Mamatha Kumari M.; Shankar M. V. Utilizing 2D materials to enhance H_2_ generation efficiency via photocatalytic reforming industrial and solid waste. Environ. Res. 2021, 200, 111239 10.1016/j.envres.2021.111239. 33992636

[ref17] Tong R.; Ng K. W.; Wang X.; Wang S.; Wang X.; Pan H. Two-dimensional materials as novel co-catalysts for efficient solar-driven hydrogen production. J. Mater. Chem. A 2020, 8 (44), 23202–23230. 10.1039/D0TA08045D.

[ref18] Shanmughan B.; Nighojkar A.; Kandasubramanian B. Exploring the future of 2D catalysts for clean and sustainable hydrogen production. Int. J. Hydrogen Energy 2023, 48 (74), 28679–28693. 10.1016/j.ijhydene.2023.04.053.

[ref19] Xiang Q.; Yu J. Graphene-Based Photocatalysts for Hydrogen Generation. J. Phys. Chem. Lett. 2013, 4 (5), 753–759. 10.1021/jz302048d. 26281930

[ref20] Li Y.; Wang H.; Xie L.; Liang Y.; Hong G.; Dai H. MoS_2_ Nanoparticles Grown on Graphene: An Advanced Catalyst for the Hydrogen Evolution Reaction. J. Am. Chem. Soc. 2011, 133 (19), 7296–7299. 10.1021/ja201269b. 21510646

[ref21] Zheng Y.; Jiao Y.; Li L. H.; Xing T.; Chen Y.; Jaroniec M.; Qiao S. Z. Toward Design of Synergistically Active Carbon-Based Catalysts for Electrocatalytic Hydrogen Evolution. ACS Nano 2014, 8 (5), 5290–5296. 10.1021/nn501434a. 24779586 PMC4046781

[ref22] Zheng Y.; Jiao Y.; Zhu Y.; Li L. H.; Han Y.; Chen Y.; Du A.; Jaroniec M.; Qiao S. Z. Hydrogen evolution by a metal-free electrocatalyst. Nat. Commun. 2014, 5 (1), 3783 10.1038/ncomms4783. 24769657

[ref23] Deng J.; Ren P.; Deng D.; Bao X. Enhanced electron penetration through an ultrathin graphene layer for highly efficient catalysis of the hydrogen evolution reaction. Angew. Chem., Int. Ed. 2015, 54 (7), 2100–4. 10.1002/anie.201409524. 25565666

[ref24] Fei H.; Dong J.; Arellano-Jiménez M. J.; Ye G.; Dong Kim N.; Samuel E. L. G.; Peng Z.; Zhu Z.; Qin F.; Bao J.; Yacaman M. J.; Ajayan P. M.; Chen D.; Tour J. M. Atomic cobalt on nitrogen-doped graphene for hydrogen generation. Nat. Commun. 2015, 6 (1), 8668 10.1038/ncomms9668. 26487368 PMC4639894

[ref25] Jiao Y.; Zheng Y.; Davey K.; Qiao S.-Z. Activity origin and catalyst design principles for electrocatalytic hydrogen evolution on heteroatom-doped graphene. Nat. Energy 2016, 1 (10), 16130 10.1038/nenergy.2016.130.

[ref26] Li J.-S.; Wang Y.; Liu C.-H.; Li S.-L.; Wang Y.-G.; Dong L.-Z.; Dai Z.-H.; Li Y.-F.; Lan Y.-Q. Coupled molybdenum carbide and reduced graphene oxide electrocatalysts for efficient hydrogen evolution. Nat. Commun. 2016, 7 (1), 11204 10.1038/ncomms11204. 27032372 PMC4822009

[ref27] Xu Y.; Wang R.; Wang J.; Li J.; Jiao T.; Liu Z. Facile fabrication of molybdenum compounds (Mo_2_C, MoP and MoS_2_) nanoclusters supported on N-doped reduced graphene oxide for highly efficient hydrogen evolution reaction over broad pH range. J. Chem. Eng. 2021, 417, 129233 10.1016/j.cej.2021.129233.

[ref28] Li Q.; Guo B.; Yu J.; Ran J.; Zhang B.; Yan H.; Gong J. R. Highly Efficient Visible-Light-Driven Photocatalytic Hydrogen Production of CdS-Cluster-Decorated Graphene Nanosheets. J. Am. Chem. Soc. 2011, 133 (28), 10878–10884. 10.1021/ja2025454. 21639097

[ref29] Stylianakis M. M.; Konios D.; Viskadouros G.; Vernardou D.; Katsarakis N.; Koudoumas E.; Anastasiadis S. H.; Stratakis E.; Kymakis E. Ternary organic solar cells incorporating zinc phthalocyanine with improved performance exceeding 8.5%. Dyes Pigm. 2017, 146, 408–413. 10.1016/j.dyepig.2017.07.032.

[ref30] Monteiro A. R.; Neves M. G. P. M. S.; Trindade T. Functionalization of Graphene Oxide with Porphyrins: Synthetic Routes and Biological Applications. ChemPlusChem. 2020, 85 (8), 1857–1880. 10.1002/cplu.202000455. 32845088

[ref31] Castro-Cruz H. M.; Macías-Ruvalcaba N. A. Porphyrin-catalyzed electrochemical hydrogen evolution reaction. Metal-centered and ligand-centered mechanisms. Coord. Chem. Rev. 2022, 458, 214430 10.1016/j.ccr.2022.214430.

[ref32] Choi W. I.; Wood B. C.; Schwegler E.; Ogitsu T. Combinatorial Search for High-Activity Hydrogen Catalysts Based on Transition-Metal-Embedded Graphitic Carbons. Adv. Energy Mater. 2015, 5 (23), 1501423 10.1002/aenm.201501423.

[ref33] Baby A.; Trovato L.; Di Valentin C. Single Atom Catalysts (SAC) trapped in defective and nitrogen-doped graphene supported on metal substrates. Carbon 2021, 174, 772–788. 10.1016/j.carbon.2020.12.045.

[ref34] Beyene B. B.; Hung C.-H. Recent progress on metalloporphyrin-based hydrogen evolution catalysis. Coord. Chem. Rev. 2020, 410, 213234 10.1016/j.ccr.2020.213234.

[ref35] O’Neill J. S.; Kearney L.; Brandon M. P.; Pryce M. T. Design components of porphyrin-based photocatalytic hydrogen evolution systems: A review. Coord. Chem. Rev. 2022, 467, 214599 10.1016/j.ccr.2022.214599.

[ref36] Dasler D.; Schäfer R. A.; Minameyer M. B.; Hitzenberger J. F.; Hauke F.; Drewello T.; Hirsch A. Direct Covalent Coupling of Porphyrins to Graphene. J. Am. Chem. Soc. 2017, 139 (34), 11760–11765. 10.1021/jacs.7b04122. 28762268

[ref37] Canton-Vitoria R.; Alsaleh A. Z.; Rotas G.; Nakanishi Y.; Shinohara H.; D’ Souza F.; Tagmatarchis N. Graphene performs the role of an electron donor in covalently interfaced porphyrin-boron azadipyrromethene dyads and manages photoinduced charge-transfer processes. Nanoscale 2022, 14 (40), 15060–15072. 10.1039/D2NR03740H. 36200654

[ref38] Chen Y.; Fu Y.; Peng W.; Wang S. Minireview of Coupled Electrochemical Hydrogen Production and Organic-Oxidation for Low Energy Consumption. Energy Fuels 2023, 37 (23), 17915–17931. 10.1021/acs.energyfuels.3c02539.

[ref39] Suguro T.; Kishimoto F.; Takanabe K. Photocatalytic Hydrogen Production under Water Vapor Feeding—A Minireview. Energy Fuels 2022, 36 (16), 8978–8994. 10.1021/acs.energyfuels.2c01478.

[ref40] Lokesh S.; Srivastava R. Advanced Two-Dimensional Materials for Green Hydrogen Generation: Strategies toward Corrosion Resistance Seawater Electrolysis—Review and Future Perspectives. Energy Fuels 2022, 36 (22), 13417–13450. 10.1021/acs.energyfuels.2c02013.

[ref41] Ishaq H.; Dincer I.; Crawford C. A review on hydrogen production and utilization: Challenges and opportunities. Int. J. Hydrogen Energy 2022, 47 (62), 26238–26264. 10.1016/j.ijhydene.2021.11.149.

[ref42] Mohd Shah N. R. A.; Mohamad Yunus R.; Rosman N. N.; Wong W. Y.; Arifin K.; Jeffery Minggu L. Current progress on 3D graphene-based photocatalysts: From synthesis to photocatalytic hydrogen production. Int. J. Hydrog. Energy 2021, 46 (14), 9324–9340. 10.1016/j.ijhydene.2020.12.089.

[ref43] Megía P. J.; Vizcaíno A. J.; Calles J. A.; Carrero A. Hydrogen Production Technologies: From Fossil Fuels toward Renewable Sources. A Mini Review. Energy Fuels 2021, 35 (20), 16403–16415. 10.1021/acs.energyfuels.1c02501.

[ref44] Dutta S. Review on Solar Hydrogen: Its Prospects and Limitations. Energy Fuels 2021, 35 (15), 11613–11639. 10.1021/acs.energyfuels.1c00823.

[ref45] Huang H.; Yan M.; Yang C.; He H.; Jiang Q.; Yang L.; Lu Z.; Sun Z.; Xu X.; Bando Y.; Yamauchi Y. Graphene Nanoarchitectonics: Recent Advances in Graphene-Based Electrocatalysts for Hydrogen Evolution Reaction. Adv. Mater. 2019, 31 (48), 1903415 10.1002/adma.201903415. 31496036

[ref46] Andreiadis E. S.; Jacques P.-A.; Tran P. D.; Leyris A.; Chavarot-Kerlidou M.; Jousselme B.; Matheron M.; Pécaut J.; Palacin S.; Fontecave M.; Artero V. Molecular engineering of a cobalt-based electrocatalytic nanomaterial for H_2_ evolution under fully aqueous conditions. Nat. Chem. 2013, 5 (1), 48–53. 10.1038/nchem.1481. 23247177

[ref47] Wang B.; Yang F.; Feng L. Recent Advances in Co-Based Electrocatalysts for Hydrogen Evolution Reaction. Small 2023, 19 (45), 2302866 10.1002/smll.202302866. 37434101

[ref48] Artero V.; Chavarot-Kerlidou M.; Fontecave M. Splitting Water with Cobalt. Angew. Chem., Int. Ed. 2011, 50 (32), 7238–7266. 10.1002/anie.201007987. 21748828

[ref49] Huang D.; Lu J.; Li S.; Luo Y.; Zhao C.; Hu B.; Wang M.; Shen Y. Fabrication of cobalt porphyrin. Electrochemically reduced graphene oxide hybrid films for electrocatalytic hydrogen evolution in aqueous solution. Langmuir 2014, 30 (23), 6990–8. 10.1021/la501052m. 24856539

[ref50] Ma J.; Liu L.; Chen Q.; Yang M.; Wang D.; Tong Z.; Chen Z. A facile approach to prepare crumpled CoTMPyP/electrochemically reduced graphene oxide nanohybrid as an efficient electrocatalyst for hydrogen evolution reaction. Appl. Surf. Sci. 2017, 399, 535–541. 10.1016/j.apsusc.2016.12.070.

[ref51] Li X.; Lei H.; Guo X.; Zhao X.; Ding S.; Gao X.; Zhang W.; Cao R. Graphene-Supported Pyrene-Modified Cobalt Corrole with Axial Triphenylphosphine for Enhanced Hydrogen Evolution in pH 0–14 Aqueous Solutions. ChemSusChem 2017, 10 (22), 4632–4641. 10.1002/cssc.201701196. 28772058

[ref52] Canales C.; Varas-Concha F.; Mallouk T. E.; Ramírez G. Enhanced electrocatalytic hydrogen evolution reaction: Supramolecular assemblies of metalloporphyrins on glassy carbon electrodes. Appl. Catal., B 2016, 188, 169–176. 10.1016/j.apcatb.2016.01.066.

[ref53] Seo S.; Lee K.; Min M.; Cho Y.; Kim M.; Lee H. A molecular approach to an electrocatalytic hydrogen evolution reaction on single-layer graphene. Nanoscale 2017, 9 (11), 3969–3979. 10.1039/C6NR09428G. 28266680

[ref54] Wang A.; Shen X.; Wang Q.; Cheng L.; Zhu W.; Shang D.; Song Y. Boosted charge transfer in porphyrin and zinc phthalocyanine co-functionalized graphene oxide nanohybrids toward improved optical limiting and H_2_ evolution. Dyes Pigm. 2021, 187, 109142 10.1016/j.dyepig.2021.109142.

[ref55] Liu J.; Bo X.; Li M.; Yin D.; Guo L. Contrastive study on porphyrinic iron metal–organic framework supported on various carbon matrices as efficient electrocatalysts. J. Colloid Interface Sci. 2018, 513, 438–447. 10.1016/j.jcis.2017.11.028. 29175737

[ref56] Aoudi B.; Khaligh A.; Sheidaei Y.; Tuncel D. In situ-Electrochemically reduced graphene oxide integrated with cross-linked supramolecular polymeric network for electrocatalytic hydrogen evaluation reaction. Polymer 2021, 231, 124140 10.1016/j.polymer.2021.124140.

[ref57] Wang A.; Shen X.; Wang Q.; Cheng L.; Zhu W.; Shang D.; Song Y. Enhanced optical limiting and hydrogen evolution of graphene oxide nanohybrids covalently functionalized by covalent organic polymer based on porphyrin. Dalton Trans. 2021, 50 (20), 7007–7016. 10.1039/D1DT00756D. 33949532

[ref58] Sahabudeen H.; Qi H.; Glatz B. A.; Tranca D.; Dong R.; Hou Y.; Zhang T.; Kuttner C.; Lehnert T.; Seifert G.; Kaiser U.; Fery A.; Zheng Z.; Feng X. Wafer-sized multifunctional polyimine-based two-dimensional conjugated polymers with high mechanical stiffness. Nat. Commun. 2016, 7, 13461 10.1038/ncomms13461. 27849053 PMC5116084

[ref59] Zhu M.; Li Z.; Xiao B.; Lu Y.; Du Y.; Yang P.; Wang X. Surfactant assistance in improvement of photocatalytic hydrogen production with the porphyrin noncovalently functionalized graphene nanocomposite. ACS Appl. Mater. Interfaces 2013, 5 (5), 1732–1740. 10.1021/am302912v. 23384090

[ref60] Yuan Y.-J.; Chen D.; Zhong J.; Yang L.-X.; Wang J.-J.; Yu Z.-T.; Zou Z.-G. Construction of a noble-metal-free photocatalytic H_2_ evolution system using MoS_2_/reduced graphene oxide catalyst and zinc porphyrin photosensitizer. J. Phys. Chem. C 2017, 121 (39), 24452 10.1021/acs.jpcc.7b08290.

[ref61] Ge R.; Li X.; Kang S. Z.; Qin L.; Li G. Highly efficient graphene oxide/porphyrin photocatalysts for hydrogen evolution and the interfacial electron transfer. Appl. Catal., B 2016, 187, 67–74. 10.1016/j.apcatb.2016.01.024.

[ref62] Luo Q.; Zhu K.; Kang S. Z.; Qin L.; Han S.; Li G.; Li X. A novel cobalt ion implanted pyridylporphyrin/graphene oxide assembly for enhanced photocatalytic hydrogen production. J. Porphyr. Phthalocyanines 2018, 22 (9–10), 877–885. 10.1142/S1088424618500785.

[ref63] Zhu K.; Luo Q.; Kang S. Z.; Qin L.; Li G.; Li X. The study of a novel cobalt-implanted pyridylporphyrin/graphene oxide nanohybrid for enhanced photocatalytic hydrogen evolution and its electron transfer mechanism. Nanoscale 2018, 10 (39), 18635–18641. 10.1039/C8NR06138F. 30259946

[ref64] Zhang L.; Qin L.; Kang S. Z.; Li G. D.; Li X. Graphene/Pyridylporphyrin Hybrids Interfacially Linked with Rare Earth Ions for Enhanced Photocatalytic Hydrogen Evolution. ACS Sustainable Chem. Eng. 2019, 7 (9), 8358–8366. 10.1021/acssuschemeng.8b06867.

[ref65] Fan Z.; Nomura K.; Zhu M.; Li X.; Xue J.; Majima T.; Osakada Y. Synthesis and photocatalytic activity of ultrathin two-dimensional porphyrin nanodisks via covalent organic framework exfoliation. Commun. Chem. 2019, 2 (1), 55 10.1038/s42004-019-0158-8.

[ref66] Li R. X.; Liu X. F.; Liu T.; Yin Y. B.; Zhou Y.; Mei S. K.; Yan J. Electrocatalytic properties of [FeFe]-hydrogenases models and visible-light-driven hydrogen evolution efficiency promotion with porphyrin functionalized graphene nanocomposite. Electrochim. Acta 2017, 237, 207–216. 10.1016/j.electacta.2017.03.216.

[ref67] Liu X. F.; Li R. X.; Ren X. T.; Yin Y. B.; Mei S. K.; Liu T.; Yan J. Synthesis of bio-inspired mononuclear nickel hydrogen production catalysts and photocatalytic efficiency improvement with porphyrin covalently functionalized graphene nanohybrid. J. Catal. 2017, 348, 314–320. 10.1016/j.jcat.2016.12.014.

[ref68] Li X.; Li K.; Wang D.; Huang J.; Zhang C.; Du Y.; Yang P. One-pot synthesis of manganese porphyrin covalently functionalized graphene oxide for enhanced photocatalytic hydrogen evolution. J. Porphyr. Phthalocyanines 2017, 21 (3), 179–188. 10.1142/S1088424616501236.

[ref69] Luo Q.; Ge R.; Kang S. Z.; Qin L.; Li G.; Li X. Fabrication mechanism and photocatalytic activity for a novel graphene oxide hybrid functionalized with tetrakis-(4-hydroxylphenyl)porphyrin and 1-pyrenesulfonic acid. Appl. Surf. Sci. 2018, 427, 15–23. 10.1016/j.apsusc.2017.08.152.

[ref70] Trauzettel B.; Bulaev D. V.; Loss D.; Burkard G. Spin qubits in graphene quantum dots. Nat. Phys. 2007, 3 (3), 192–196. 10.1038/nphys544.

[ref71] Ji Y.; Zuo Q.; Chen C.; Liu Y.; Mai Y.; Zhou Y. A supramolecular single-site photocatalyst based on multi-to-one Forster resonance energy transfer. Chem. Commun. 2021, 57 (34), 4174–4177. 10.1039/D1CC01339D. 33908478

[ref72] Liu Y.; Jin J.; Deng H.; Li K.; Zheng Y.; Yu C.; Zhou Y. Protein-Framed Multi-Porphyrin Micelles for a Hybrid Natural–Artificial Light-Harvesting Nanosystem. Angew. Chem., Int. Ed. 2016, 55 (28), 7952–7957. 10.1002/anie.201601516. 27187799

[ref73] Liu Z.; Chen Y.; Zhang T.; Qin L.; Kang S.-Z.; Li X. Interfacial construction of porphyrin nanorods synergistically regulated by graphene and copper anchored onto carbon nitride for boosted photocatalytic hydrogen generation. Ceram. Int. 2023, 49 (5), 8390–8397. 10.1016/j.ceramint.2022.10.371.

[ref74] Zeng R.; Chen G.; Xiong C.; Li G.; Zheng Y.; Chen J.; Long Y.; Chen S. Room temperature Zinc-metallation of cationic porphyrin at graphene surface and enhanced photoelectrocatalytic activity. Appl. Surf. Sci. 2018, 434, 756–762. 10.1016/j.apsusc.2017.10.206.

[ref75] Sahar K. U.; Rafiq K.; Abid M. Z.; Rehman U. U.; Althomali R. H.; Rauf A.; Hussain E. Sensitization of TiO_2_/g-C_3_N_4_ Heterostructures via Pd–Au Cocatalysts: A Rational Design of Water Splitting System for Green Fuel Production. Energy Fuels 2024, 10.1021/acs.energyfuels.4c01320.

[ref76] Wang P.; Zhang X.; Shi R.; Zhao J.; Yuan Z.; Zhang T. Light-Driven Hydrogen Production from Steam Methane Reforming via Bimetallic PdNi Catalysts Derived from Layered Double Hydroxide Nanosheets. Energy Fuels 2022, 36 (19), 11627–11635. 10.1021/acs.energyfuels.2c01349.

[ref77] Magnera T. F.; Dron P. I.; Bozzone J. P.; Jovanovic M.; Rončević I.; Tortorici E.; Bu W.; Miller E. M.; Rogers C. T.; Michl J. Porphene and porphite as porphyrin analogs of graphene and graphite. Nat. Commun. 2023, 14 (1), 6308 10.1038/s41467-023-41461-w. 37813887 PMC10562370

[ref78] Pavlak I.; Matasović L.; Buchanan E. A.; Michl J.; Rončević I. Electronic Structure of Metalloporphenes, Antiaromatic Analogues of Graphene. J. Am. Chem. Soc. 2024, 146 (6), 3992–4000. 10.1021/jacs.3c12079. 38294407 PMC10870706

[ref79] Scalfi L.; Becker M. R.; Netz R. R.; Bocquet M.-L. Enhanced interfacial water dissociation on a hydrated iron porphyrin single-atom catalyst in graphene. Commun. Chem. 2023, 6 (1), 236 10.1038/s42004-023-01027-9. 37919471 PMC10622426

